# Chondroprotection of PPARα activation by WY14643 via autophagy involving Akt and ERK in LPS‐treated mouse chondrocytes and osteoarthritis model

**DOI:** 10.1111/jcmm.14184

**Published:** 2019-02-07

**Authors:** Yang Zhou, Xiaolei Chen, Ning Qu, Bing Zhang, Chun Xia

**Affiliations:** ^1^ Zhongshan Hospital, Xiamen University Xiamen Fujian China; ^2^ School of Medicine Xiamen University Xiamen Fujian China

**Keywords:** autophagy, mouse OA model, OA chondrocyte, PPARα, WY14643

## Abstract

Autophagy maintains cellular homoeostasis. The enhancement of autophagy in chondrocytes could prevent osteoarthritis (OA) progression in articular cartilage. Peroxisome proliferator‐activated receptor α (PPARα) activation may also protect articular chondrocytes against cartilage degradation in OA. However, whether the protective effect of activated PPARα is associated with autophagy induction in chondrocytes is not determined. In this study, we investigated the effect of PPARα activation by its agonist, WY14643, on the protein expression level of Aggrecan and ADAMTS5, and the protein expression level of autophagy biomarkers, including LC3B and P62, using Western blotting analysis in isolated mouse chondrocytes pre‐treated with lipopolysaccharides (LPS, mimicking OA chondrocytes) with or without the autophagy inhibitor chloroquine diphosphate salt. Furthermore, Akt and ERK phosphorylation was detected in LPS‐treated chondrocytes in response to WY14643. In addition, the effect of intra‐articularly injected WY14643 on articular cartilage in a mouse OA model established by the destabilization of the medial meniscus was assessed using the Osteoarthritis Research Society International (OARSI) histopathology assessment system, along with the detection of Aggrecan, ADAMTS5, LC3B and P62 protein levels using immunohistochemistry assay. The results indicated that PPARα activation by WY14643 promoted proteoglycan synthesis by autophagy enhancement in OA chondrocytes in vivo and in vitro concomitant with the elevation of Akt and ERK phosphorylation. Therefore, autophagy could contribute to the chondroprotection of PPARα activation by WY14643, with the implication that PPARα activation by WY14643 may be a potential approach for OA therapy.

## 1 INTRODUCTION

Osteoarthritis (OA) is a degenerative joint disease, characterized by extracellular matrix (ECM) damage and chondrocyte death. Proteoglycan aggregates and collagen fibrils are major components of ECM. Several lines of evidence indicate that the proteoglycan biosynthetic capacity or expression of anabolic genes is decreased in chondrocytes of OA patients.^1^, ^2^, ^3^ Improvement of proteoglycan biosynthesis is an effective therapeutic approach for OA.^4^ Apple procyanidins are promising food components that inhibit OA progression by promoting mitochondrial biogenesis and proteoglycan homoeostasis with the up‐regulation of Aggrecan in primary chondrocytes.^5^ FoxO transcription factors modulate proteoglycan in cartilage homoeostasis and OA, protecting against OA‐associated cartilage damage.^6^ Suramin increases cartilage proteoglycan accumulation in vitro and protects against joint damage triggered by papain injection in mouse knees in vivo.^7^

As one of three subtypes of peroxisome proliferator‐activated receptors (PPARs, including PPARα，PPARβ/δ and PPARγ), PPARα responds to specific ligands by altering gene expression to regulate lipid and lipoprotein metabolism, apoptosis and inflammatory responses in the liver and other organs of the human body.^8^, ^9^, ^10^ Some studies have reported that PPARα plays an important role in chondrocyte metabolism. Activation of PPARs α, β/δ and γ impairs TGF‐β1‐induced collagen production and modulates the TIMP‐1/MMPs balance in three‐dimensional cultured chondrocytes.^11^ Agonists of PPARα, β/δ and γ reduce TGF‐β1‐induced proteoglycans' production in chondrocytes.^12^ PPARα down‐regulates AGE‐induced TGF‐β and MMP‐9 expression in chondrocytes.^13^ PPARα activation pathway potentiates interleukin‐1 receptor antagonist production in cytokine‐treated chondrocytes.^14^ In 2011, Clockaerts et al report that PPARα activation by its agonist, WY14643, decreases inflammatory and destructive responses in OA cartilage, although it does not have an effect on COL2A1 or Aggrecan mRNA expression.^15^ These studies indicate that PPARα activation may have a protective effect on articular cartilage against OA progression.

Autophagy is a highly conserved homoeostatic process, maintaining cellular homoeostasis by sequestering and degrading cytosolic macromolecules, damaged organelles and some pathogens.^16^ Moreover, the relationship between PPARα and autophagy and its effect on cell metabolism have been reported.^9^, ^17^, ^18^, ^19^ TAK1 regulates hepatic lipid metabolism and tumorigenesis via the AMPK/mTORC1 axis, affecting both autophagy and PPARα activity.^17^ Inhibition of glycogen synthase kinase 3β promotes autophagy to protect mice from acute liver failure (ALF) mediated by PPARα.^18^ PPARα‐mediated induction of autophagy ameliorates liver injury in cases of ALF by attenuating inflammatory responses, indicating a potential therapeutic application for ALF treatment.^9^ Pharmacologic activation of PPARα reverses the normal suppression of autophagy in the fed state, inducing autophagic lipid degradation, or lipophagy.^19^ These studies demonstrate that PPARα could induce autophagy to protect liver against pathological damage. Lots of evidence indicates that autophagy also participates in OA pathological progression and the enhancement of autophagy could protect articular cartilage from OA progression.^20^, ^21^, ^22^, ^23^ For example, autophagy is a protective mechanism in normal cartilage, and its ageing‐related loss is linked with cell death and OA.^20^ Autophagy activation by rapamycin and Torin 1 reduces severity of experimental OA.^21^, ^22^, ^23^ However, whether PPARα might regulate autophagy to protect cartilage against OA pathological progression is not determined.

In this study, we detected the effect of PPARα activation by its agonist WY14643 on autophagy and proteoglycan synthesis in LPS‐treated mouse articular chondrocytes (mimicking OA chondrocytes)^24^, ^25^ and OA model established by the destabilization of the medial meniscus (DMM). Our findings showed that PPARα activation by WY14643 promoted proteoglycan synthesis by the enhancement of autophagy in OA chondrocytes in vivo and in vitro concomitant with the elevation of Akt and ERK phosphorylation, with the implication that WY14643 may be a potential approach for OA therapy.

## 2 MATERIALS AND METHODS

### 2.1 Reagents and antibodies

Antibodies against PPARα, ADAMTS5 and P62 were purchased from Abcam Inc (Cambridge, MA, USA). Antibodies against Akt, p‐Akt(Ser473), ERK, p‐ERK(Thr202/Tyr204) and MEK inhibitor PD98059 were purchased from Cell Signaling Technology Inc (Beverly, MA, USA). Antibodies against Aggrecan, PPARα inhibitor WY14643, Akt inhibitor Triciribine (TCN), autophagy inhibitor chloroquine diphosphate salt (CQ) and LPS were purchased from Sigma‐Aldrich in China (Shanghai, China). Antibodies against LC3B and β‐actin were obtained from Novus Biologicals (Littleton, Colorado, USA) and Shanghai Abways Biotechnology (Shanghai, China) respectively. Other reagents of the highest grade were commercially available.

### 2.2 Mouse chondrocyte isolation and culture

Chondrocytes of knee cartilage were isolated from neonatal male C57BL/6J mice (within 24‐72 hours after birth), after approval of the committee on the Ethics of Animal Experiments of Xiamen University (ID no. 20170301). Primary chondrocytes were cultured in DMEM containing 10% foetal bovine serum to 80 confluence and plated in 60‐mm cell culture dishes for the subsequent experiments as previously described.^20^

### 2.3 Western blotting analysis

Protein extracts were subjected to SDS‐PAGE (8%‐12%) and transferred to a PVDF membrane (GE Healthcare, Herfordshire, UK) as previously described.^26^, ^27^ The membrane was incubated with various primary antibodies as required at 4°C overnight, followed by the addition of the corresponding secondary antibodies at room temperature for 1‐2 hours. An enhanced chemiluminescence detection kit was used to detect antibody reactivity (Pierce, Rockford, IL, USA).

### 2.4 Established mouse model of experimental OA

Six‐week‐old male mice (average weight 28 g) were acclimatized to the laboratory environment for 2 weeks before the following experiments. Mouse joints were randomly divided into four groups (16 mice, 32 knee joints), including sham (four mice, eight knee joints), control (OA, four mice, eight knee joints), OA+DMSO (four mice, eight knee joints), and OA+WY14643 (four mice, eight knee joints). The mouse experimental model of OA was induced by DMM as previously described.^28^ After the first week post‐surgery, DMSO and WY14643 (5 × 10^−3^ g/kg) were intra‐articularly injected once every 3 days, respectively, and mice were not killed until 4 weeks after injection. The protocol was approved by the committee on the Ethics of Animal Experiments of Xiamen University (ID no. 20170301).

### 2.5 Histopathological scores

The fresh samples were fixed in 4% paraformaldehyde for 48 hours followed by decalcification in 10% EDTA‐2Na for 3 weeks, and then paraffin‐embedded. Three‐micrometre‐thick sections of the samples were cut in sagittal plane from the medial side of joint and were stained by Safranin O‐Fast green stain. The articular cartilage thickness in medial femur condyle and tibial plateau (from superficial zone to subchondral bone) was measured using Image‐Pro Plus 6.0 software by the two blinded observers. The degree of articular cartilage degeneration was then assessed using the OARSI histopathology assessment system.^29^ Grade 0 in OARSI system represents normal cartilage and increasing grade (from 1 to 6) indicates a more biologically cartilage degeneration.

### 2.6 Immunohistochemistry assay

Three‐micrometre‐thick sections were obtained from the paraffin‐embedded specimens prior to immunohistochemistry assay. According to the manufacturer's instruction (MAIXIN.BIO, Fuzhou, China), the sections were incubated overnight at 4°C with primary antibody: Aggrecan (1:800), ADAMTS5 (1:400), p‐Akt and p‐ERK (1:100), LC3B and P62 (1:200) dilutions, respectively, and secondary antibodies. Diaminobenzidine was used to visualize the immunohistochemical reaction followed by being counterstained with haematoxylin. Photomicrographs were taken with OLYMPUS BX41 microscope equipped with a digital camera. The number of positive chondrocytes (dark brown cells) was counted and analysed by the two blinded observers using GraphPad Prism version.^30^

### 2.7 Statistics analysis

Data were presented as means ± SEM at least three separate experiments for each group. After a test for normal distribution and Variance homogeneity detection, differences between the groups were examined for statistical significance using one‐way analysis of variance following Tukey’s post hoc tests with GraphPad Prism version 5 (GraphPad Software, Inc, San Diego, CA. USA). OARSI Scoring was performed using Kruskal‐Wallis test with spss 19.0 software (SPSS, Chicago, IL). Statistical significance was determined at level of *P* < 0.05.*P < 0.05;**P < 0.01.

## 3 RESULTS

### 3.1 PPARα activation by WY14643 promoted proteoglycan synthesis by the enhancement of autophagy in LPS‐treated mouse articular chondrocytes

**Figure 1:**
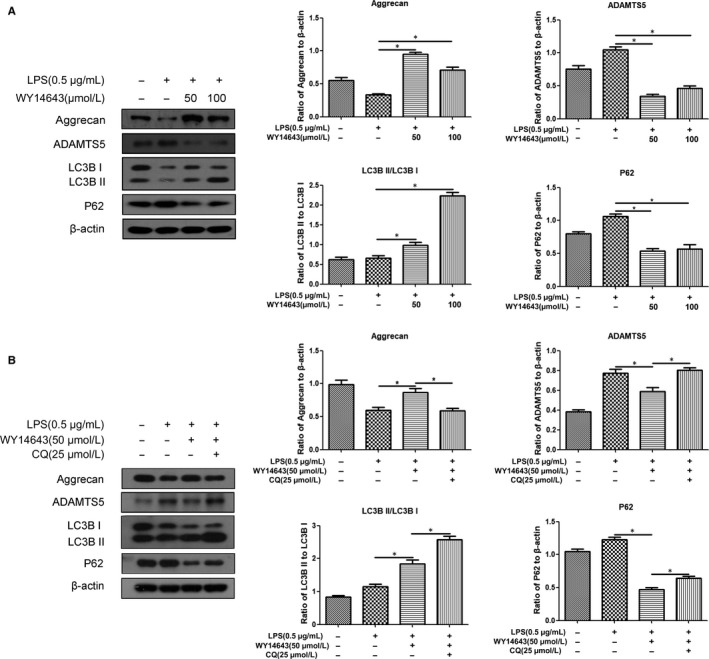
Effect of PPARα activation by WY14643 on proteoglycan synthesis and autophagy in LPS‐treated mouse articular chondrocytes. A. Cells were pre‐treated with LPS (0.5 μg/mL) for 2 h prior to the treatment of WY14643 (0, 50, 100 μmol/L) for 24 h. Aggrecan, ADAMTS5, LC3B, P62 and β‐actin protein levels were detected via Western blotting. B. Cells were pre‐treated with LPS (0.5 μg/mL) for 2 h prior to the treatment of WY14643 (50 μmol/L) with or without CQ (25 μmol/L) for 24 h. Aggrecan, ADAMTS5, LC3B, P62 and β‐actin protein levels were detected via Western blotting. The data are reported as the means ± SEM of three or five independent experiments (*P < 0.05)

To investigate whether autophagy linked to PPARα participated in OA pathological progression, the biomarkers of proteoglycan synthesis and autophagy were detected with Western blotting in LPS‐treated mouse articular chondrocytes (mimicking OA pathological condition, Figures S1 and S2) responded to WY14643, a potent and selective agonist of PPARα 9, 11 Figures S3 and S4]. As shown in Figure 1A, the addition of either 50 or 100 μmol/L of WY14643 led to a significant increase in Aggrecan protein level and a significant decrease in ADAMTS5 protein level (*P < 0.05, vs untreated group). Meanwhile, the ratio of LC3BII/I was significantly increased by WY14643 (Figure 1A，**P < 0.01, vs untreated group), along with a drastic decrease in P62 protein level (Figure1A，*P < 0.05, vs untreated group). Furthermore, the addition of CQ, an inhibitor of autophagy, partially reversed the effect of WY14643 on Aggrecan and ADAMTS5 expression (Figure 1B, *P < 0.05, vs WY14643‐treated group).

### 3.2 AKT and ERK were involved in autophagy enhancement and proteoglycan synthesis by WY14643‐stimulated PPARα activation in LPS‐treated mouse articular chondrocytes

**Figure 2:**
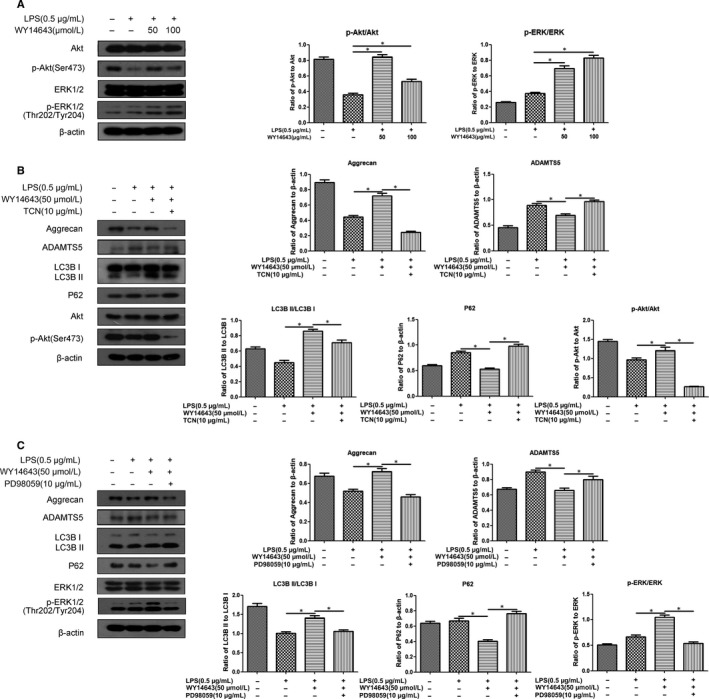
Effect of PPARα activation by WY14643 on Akt and ERK expression in LPS‐treated mouse articular chondrocytes. A. Cells were pre‐treated with LPS (0.5 μg/mL) for 2 h prior to the treatment of WY14643 (0, 50, 100 μmol/L) for 24 h. Akt, p‐Akt, ERK, p‐ERK and β‐actin protein levels were detected via Western blotting. B. Cells were pre‐treated with LPS (0.5 μg/mL) for 2 h prior to the treatment of WY14643 (50 μmol/L) with or without TCN (10 μmol/L) for 24 h. Aggrecan, ADAMTS5, LC3B, P62, Akt, p‐Akt and β‐actin protein levels were detected via Western blotting. C. Cells were pre‐treated with LPS (0.5 μg/mL) for 2 h prior to the treatment of WY14643 (50 μmol/L) with or without PD98059 (10 μmol/L) for 24 h. Aggrecan, ADAMTS5, LC3B, P62, ERK, p‐ERK and β‐actin protein levels were detected via Western blotting. The data are reported as the means ± SEM of three or five independent experiments (*P < 0.05)

As shown in Figure 2A, the addition of either 50 or 100 μmol/L WY14643 significantly elevated the phosphorylation of Akt and ERK, while total Akt and ERK did not change (*P < 0.05, vs untreated group). Furthermore, the addition of the Akt inhibitor, TCN (10 μmol/L), restored the alterations in Aggrecan and ADAMTS5 expressions induced by WY14643, along with a decrease in LC3BII/I and an increase in P62 expression level (Figure 2B, *P < 0.05,**P < 0.01, vs WY14643‐treated group). Meanwhile, the addition of the MEK inhibitor, PD98059 (10 μmol/L), also offset the effect of WY14643 on these biomarkers of proteoglycan synthesis and autophagy (Figure 2C,*P < 0.05, vs WY14643‐treated group).

### 3.3 Intra‐articular injection of WY14643 ameliorated articular cartilage degeneration in a mouse OA model

**Figure 3:**
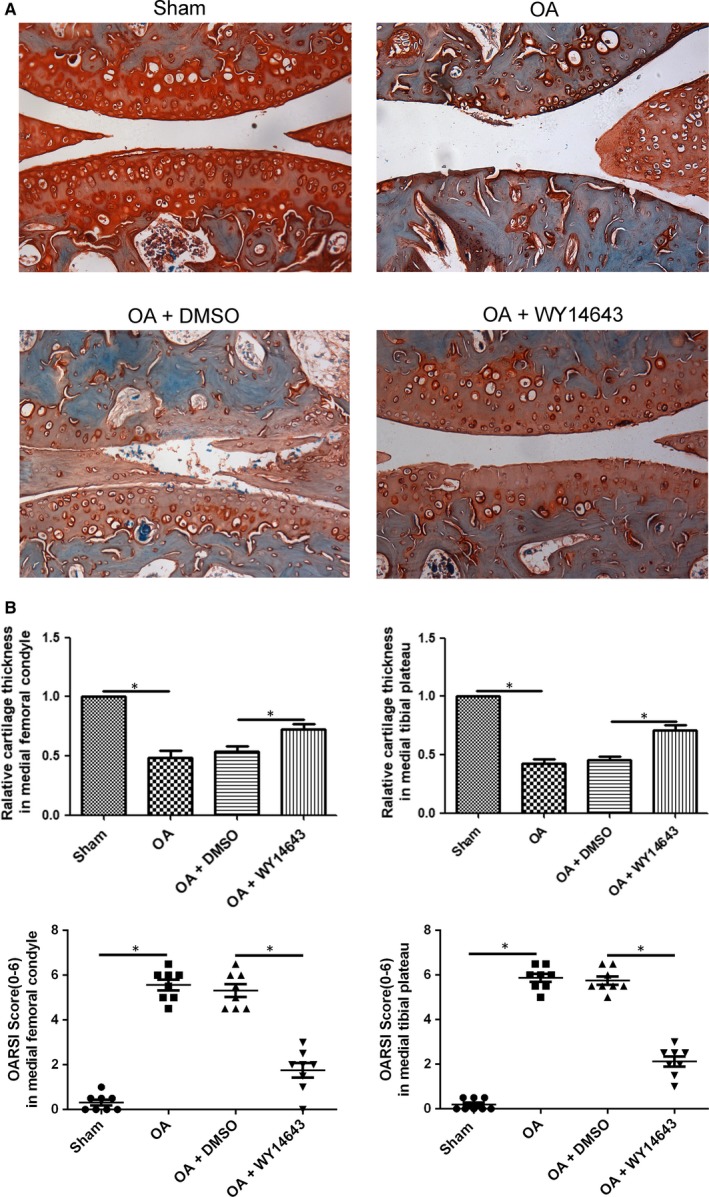
Effect of intra‐articular injection of WY14643 on articular cartilage in a mouse OA model. A. Sections in different treated groups were examined using Safranin O‐Fast green stain (original magnification ×20). B. Bar graph shows the change in articular cartilage thickness in medial femur condyle and tibial plateau. Data are mean ± SEM of eight joints per group (*P < 0.05). C. Bar graphs show the histopathological scores performed by OARSI histopathology assessment system in medial femur condyle and tibial plateau. Data are mean ± SEM of eight joints per group (*P < 0.05)

As shown in Figure 3A, the smooth cartilage surface of femur condyle and tibial plateau, normal architecture of matrix and intact and appropriate cell distribution were observed in the Sham group. The heavy degeneration of articular cartilage in OA and OA+DMSO groups was displayed in Figure 3A, including loss of articular cartilage, sparse distribution of chondrocytes and lightly stained cartilage matrix. The intra‐articular injection of WY14643 (5 × 10^−3^ g/kg) successfully ameliorated the destruction of articular cartilage, including increasing cartilage thickness in medial femur condyle and tibial plateau (from superficial zone to subchondral bone, Figure S5), matrix density and cell number (Figure 3B,*P < 0.05, vs OA+DMSO group). Meanwhile, OARSI scores of either medial femur condyle or tibial plateau significantly declined in the OA+WY14643 group, indicating that the severity of articular cartilage degeneration was ameliorated partly by intra‐articularly injected WY14643 (Figure 3C,*P < 0.05, vs OA+DMSO group).

### 3.4 Intra‐articular injection of WY14643 promoted proteoglycan synthesis in a mouse OA model

**Figure 4:**
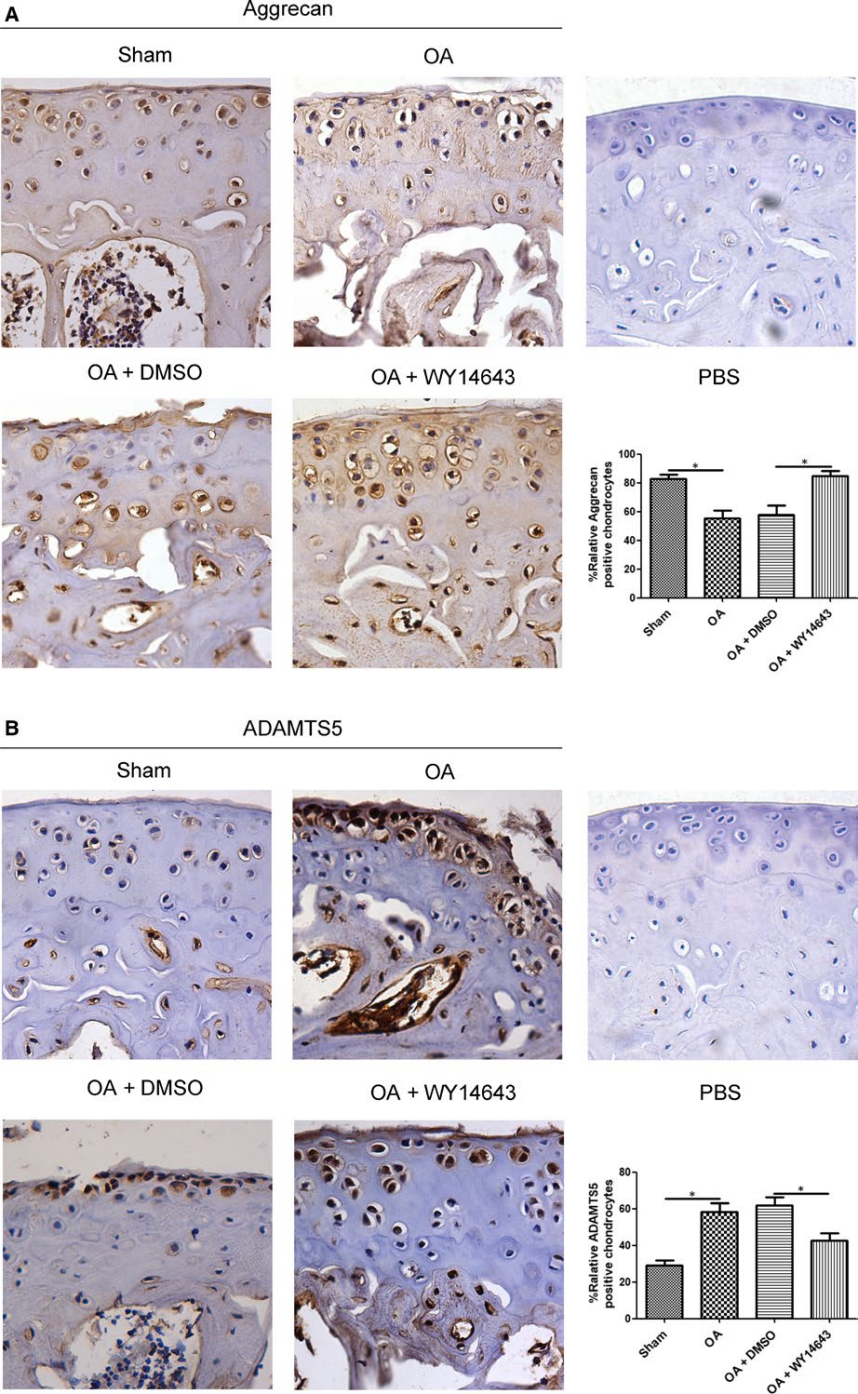
Effect of intra‐articular injection of WY14643 on Aggrecan and ADAMTS5 expressions in chondrocytes of a mouse OA model. Sections in different treated groups were examined using immunohistochemistry assay. A. Representative images from mice of different treated groups expressing Aggrecan (original magnification ×40). Bar graph shows the percentage of positive chondrocytes expressing Aggrecan. Data are mean ± SEM of 500 cells per group (*P < 0.05). B. Representative images from mice of different treated groups expressing ADAMTS5 (original magnification ×40). Bar graph shows the percentage of positive chondrocytes expressing ADAMTS5. Data are mean ± SEM of 500 cells per group (*P < 0.05)

To confirm whether WY14643 ameliorated articular cartilage degeneration via enhancing proteoglycan synthesis, Aggrecan and ADAMTS5 expression levels in chondrocytes of medial femur condyle and tibial plateau were measured using immunohistochemistry assay. As shown in Figure 4A, in Sham and OA+WY14643 groups, Aggrecan distributed mainly in cytoplasm and ECM; in OA and OA+DMSO groups, it distributed mainly in chondrocyte nucleus. Intra‐articular injection of WY14643 significantly elevated Aggrecan expression level in medial femur condyle and tibial plateau (Figure 4A,*P < 0.05, vs OA+DMSO group). In contrast with Aggrecan, ADAMTS5 expression level declined in WY14643‐treated group, compared with OA+DMSO group (Figure 4B,*P < 0.05).

### 3.5 Intra‐articular injection of WY14643 enhanced autophagy in a mouse OA model

**Figure 5:**
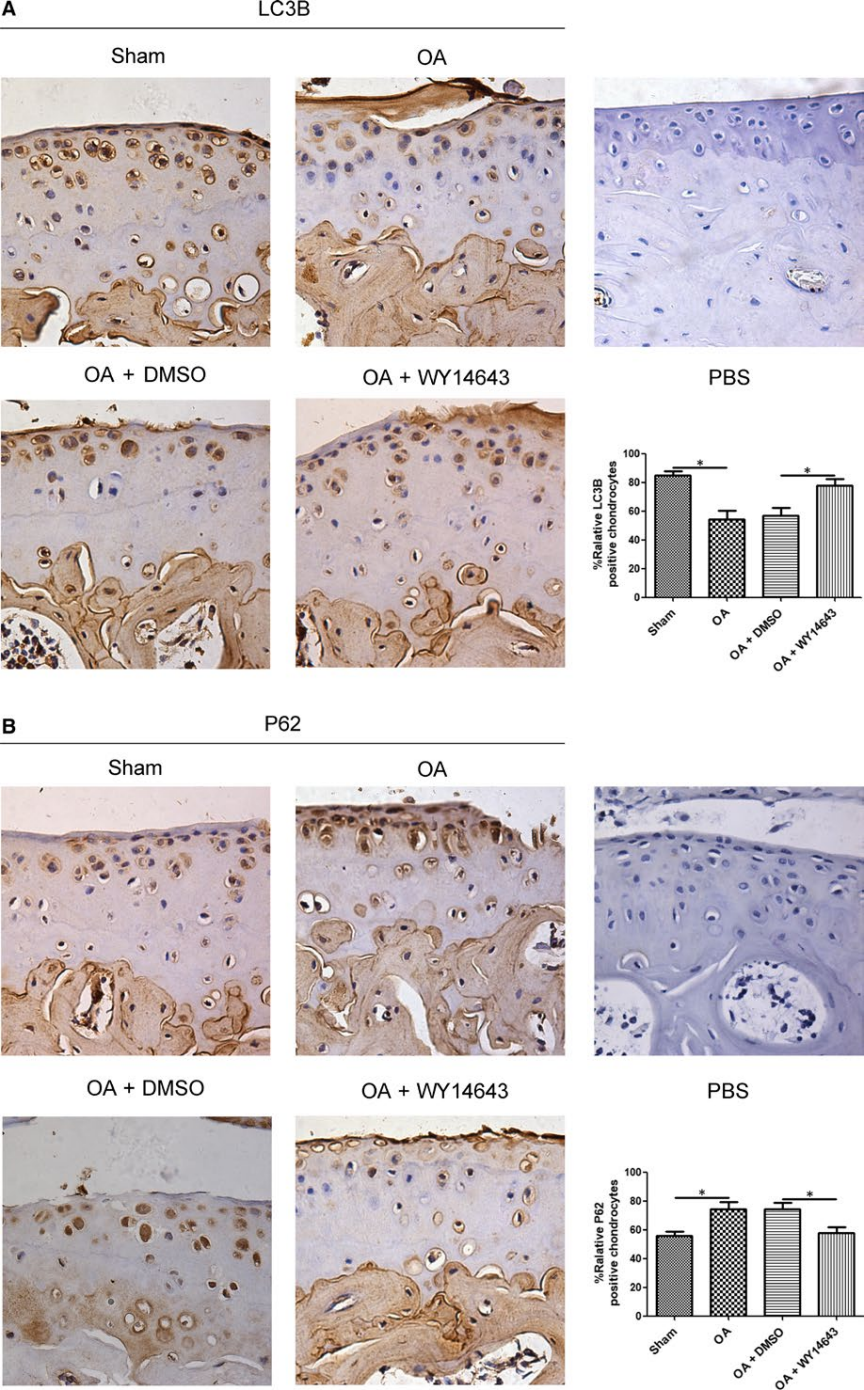
Effect of intra‐articular injection of WY14643 on LC3B and P62 expressions in chondrocytes of a mouse OA model. Sections in different treated groups were examined using immunohistochemistry assay. A. Representative images from mice of different treated groups expressing LC3B (original magnification ×40). Bar graph shows the percentage of positive chondrocytes expressing LC3B. Data are mean ± SEM of 500 cells per group (*P < 0.05). B. Representative images from mice of different treated groups expressing P62 (original magnification ×40). Bar graph shows the percentage of positive chondrocytes expressing P62. Data are mean ± SEM of 500 cells per group (*P < 0.05)

As shown in Figure 5A, the LC3B expression level in either OA or OA+DMSO group was lower than Sham and OA+WY14643 groups. Compared with OA+DMSO group, intra‐articular injection of WY14643 significantly elevated LC3B expression in OA+WY14643 group (Figure 5A,*P < 0.05). P62 expression level in OA or OA+DMSO group was higher than Sham and OA+WY14643 groups (Figure 5B). Intra‐articular injection of WY14643 significantly reduced P62 expression in OA+WY14643 group, compared with OA+DMSO group (Figure 5B, *P < 0.05).

### 3.6 Intra‐articular injection of WY14643 enhanced p‐AKT and p‐ERK in a mouse OA model

**Figure 6:**
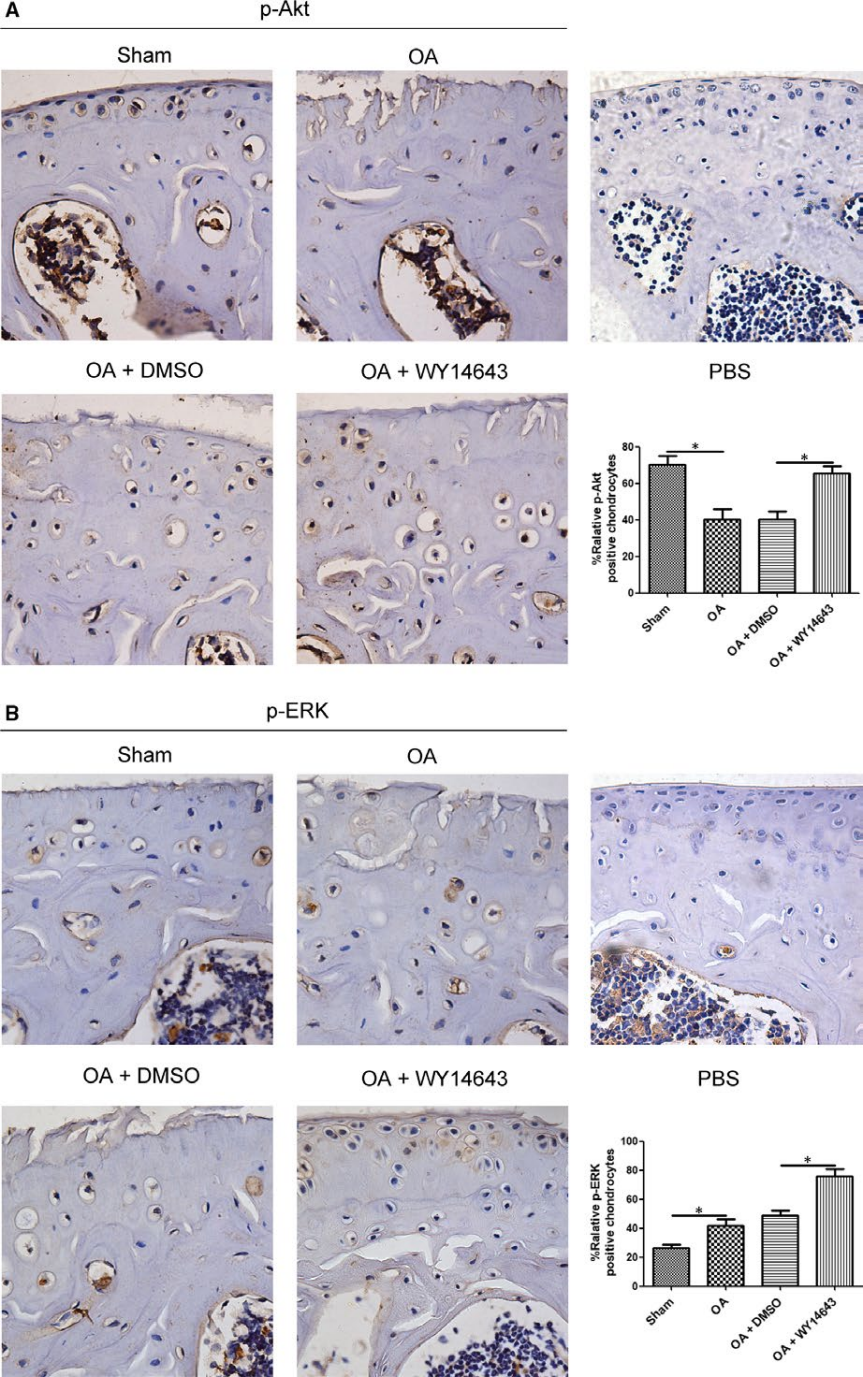
Effect of intra‐articular injection of WY14643 on p‐Akt and p‐ERK levels in chondrocytes of a mouse OA model. Sections in different treated groups were examined using immunohistochemistry assay. A. Representative images from mice of different treated groups expressing p‐Akt (original magnification ×40). Bar graph shows the percentage of positive chondrocytes expressing p‐Akt. Data are mean ± SEM of 500 cells per group (*P < 0.05). B. Representative images from mice of different treated groups expressing p‐ERK (original magnification ×40). Bar graph shows the percentage of positive chondrocytes expressing p‐ERK. Data are mean ± SEM of 500 cells per group (*P < 0.05)

As shown in Figure 6A, p‐Akt in either OA or OA+DMSO group was lower than Sham and OA+WY14643 groups. Intra‐articular injection of WY14643 significantly elevated p‐Akt level, near to Sham group (*P < 0.05, vs OA+DMSO group). P‐ERK mainly expressed at a lower level in Sham, OA and OA+DMSO groups (Figure 6B). Similar to p‐Akt, p‐ERK was significantly increased in the WY14643‐treated group, compared with OA+DMSO group (Figure 6B,*P < 0.05).

## 4 DISCUSSION

Our findings demonstrate that PPARα activation by WY14643 promoted proteoglycan synthesis via the enhancement of autophagy in LPS‐treated mouse articular chondrocytes (mimicking OA chondrocytes) concomitant with the elevation of Akt and ERK phosphorylation. Akt and ERK activation positively regulated autophagy enhancement by WY14643‐stimulated PPARα activation. Furthermore, intra‐articular injection of WY14643 ameliorated cartilage degeneration along with increased autophagy and phosphorylation of Akt and ERK in a mouse OA model established by DMM. Therefore, PPARα activation by WY14643 could ameliorate articular cartilage degeneration of OA via enhancing autophagy in mimicking OA chondrocytes in vitro and in vivo; suggesting that PPARα activation by WY14643 may be a potent approach for OA therapy.

PPARα expression is found in chondrocyte as well as PPARγ, and activated PPARα in chondrocytes can lead to structural and functional changes.^11^, ^12^, ^13^, ^14^, ^15^, ^31^ Consistent with those studies, our results showed that PPARα activation by WY14643 promoted proteoglycan synthesis in LPS‐treated mouse articular chondrocytes as well as mouse OA model established by DMM. Similarly, Huang et al have reported that PPARα agonist WY‐14643 inhibits LPS‐treated inflammation in synovial fibroblasts via NF‐kB pathway.^32^ Therefore, it is suggested that PPARα activation by WY14643 could promote proteoglycan synthesis in LPS‐treated mouse articular chondrocytes and ameliorate cartilage degeneration in a mouse OA model. Furthermore, we demonstrated that the protection by activated PPARα by WY14643 on OA chondrocyte was because of the enhancement of autophagy as the inhibition of autophagy by CQ reversed its protective effect on chondrocytes, consistent with those studies in hepatocytes.^5^, ^17^, ^18^, ^19^ Collectively, autophagy enhancement could contribute to the promoted effect of activated PPARα by WY14643 on proteoglycan synthesis in LPS‐treated chondrocytes, protecting chondrocyte against OA damage.

The positively regulatory effect of PPARα activation on Akt phosphorylation has previously been reported. PPARα activation protects the type 2 diabetic rat myocardium against ischaemia‐reperfusion injury via the activation of the PI3K/Akt and nitric oxide pathway.^33^ An enhanced Akt phosphorylation is observed in the ischaemic myocardium of WY14643‐treated rats.^34^ We also observed that WY14643 promoted Akt phosphorylation concomitant with proteoglycan synthesis in LPS‐treated chondrocyte model, while Akt inhibitor, TCN, reversed the effect of WY14643 on proteoglycan synthesis. Therefore, in LPS‐treated chondrocyte (mimicking OA chondrocyte), it is suggested that PPARα activation by WY14643 promoted proteoglycan synthesis by increasing Akt phosphorylation.

As a key regulator of mTOR (a suppressor of autophagy), Akt is always also considered to be an autophagy suppressor. For example, sucrose induces autophagy via inhibiting Akt/mTOR pathway in human chondrocytes.^35^ In OA human cartilage, increased PTEN (inhibiting Akt), AMPK and autophagy reflected the chondrocyte responses observed during starvation and steroids depletion.^36^ However, Lu et al report that rasfonin enhances autophagy with a concomitant down‐regulation of mTORC1 signalling and up‐regulation of Akt activity via glycolytic pathway.^37^ In this study, we also found that WY14643 induced autophagy concomitant with the elevation of Akt phosphorylation. TCN, an Akt inhibitor, partially reduced WY14643‐induced autophagy. It might be due to the effect of one of the upstream regulators of Akt, the class IA phosphatidylinositol 3‐kinase p110‐β subunit, which has been reported to be a positive regulator of autophagy.^38^ Hence, Akt might not consistently function as an autophagy suppressor and regulate autophagy in a context‐dependent manner,^37^ and it is conceivable that Akt activation could promote autophagy induction in WY14643‐treated OA chondrocytes.

Activation of JNK and ERK is attenuated by WY‐14643 treatment in the acute liver injury mouse model.^9^ Inactivation of PPARα results from the activation of MAPK pathway during cardiac hypertrophic growth.^39^ These studies indicate the reciprocal inhibition between PPARα and MAPK. However, PPARα ligands have been shown to activate MAPK family members in a variety of different cell types.^40^ Gardner et al have previously reported that the PPARα agonist, nafenopin, rapidly induces ERK and/or p38 phosphorylation in rat liver epithelial cells (GN4).^41^ Consistent with Gardner et al's study, we also observed that WY14643, one of the PPARα ligands, elevated ERK phosphorylation in LPS‐treated chondrocytes. The contradictory results might be because of the property of PPARα. As a nuclear receptor, PPARα controls a variety of target genes to regulate cell metabolism. Meanwhile, as a phosphoprotein, PPARα is involved in cell events in a nongenomic manner through crosstalking to lots of signal molecules.^8^, ^40^ Therefore, we speculated that the elevation of ERK phosphorylation by WY14643 in LPS‐treated chondrocytes might be as a result of the crosstalk among PPARα, NF‐κB and ERK as well as previous studies in other cells.^9^, ^42^, ^43^, ^44^

Cisplatin treatment activates ERK and subsequently promotes autophagy in ovarian cancer cells.^45^ Inhibition of MEK/ERK activation attenuates autophagy and potentiates pemetrexed‐induced activity against HepG2 hepatocellular carcinoma cells.^46^ Platycodin D induces apoptosis and triggers ERK‐ and JNK‐mediated autophagy in human hepatocellular carcinoma BEL‐7402 cells.^47^ Consistent with the aforementioned studies, our data that PD98059, a MAPK inhibitor, partially reduced autophagy enhancement by WY14643‐stimulated PPARα activation indicate the promoted effect of p‐ERK on autophagy in LPS‐treated chondrocytes. Therefore, we suggest that PPARα activation by WY14643 enhanced autophagy through the elevation of ERK phosphorylation in LPS‐treated chondrocytes.

The PPARα agonists, such as fibrates, fenofibrate, could effectively ameliorate OA symptoms in cell, animal OA model and OA patients. For example, fibrates modulate inflammatory properties of IPFP (infrapatellar fat pad) and synovium, representing a potential disease‐modifying drug for OA therapy.^48^ In erosive hand OA patients, fenofibrate treatment was associated with significant decreases in pain score, tender joint count, duration of morning stiffness, disease activity score, Cochin index and ESR.^49^ In this study, we found that WY14643 ameliorated cartilage degeneration in a mouse OA model concomitant with autophagy enhancement. Similarly, WY14643 inhibits LPS‐induced inflammation in synovial fibroblasts via NF‐kB pathway 32 and inhibits the inflammatory and destructive responses in human OA cartilage explants.^15^ Hence, WY14643 may also ameliorate OA symptoms like other PPARα agonists.

**Figure 7:**
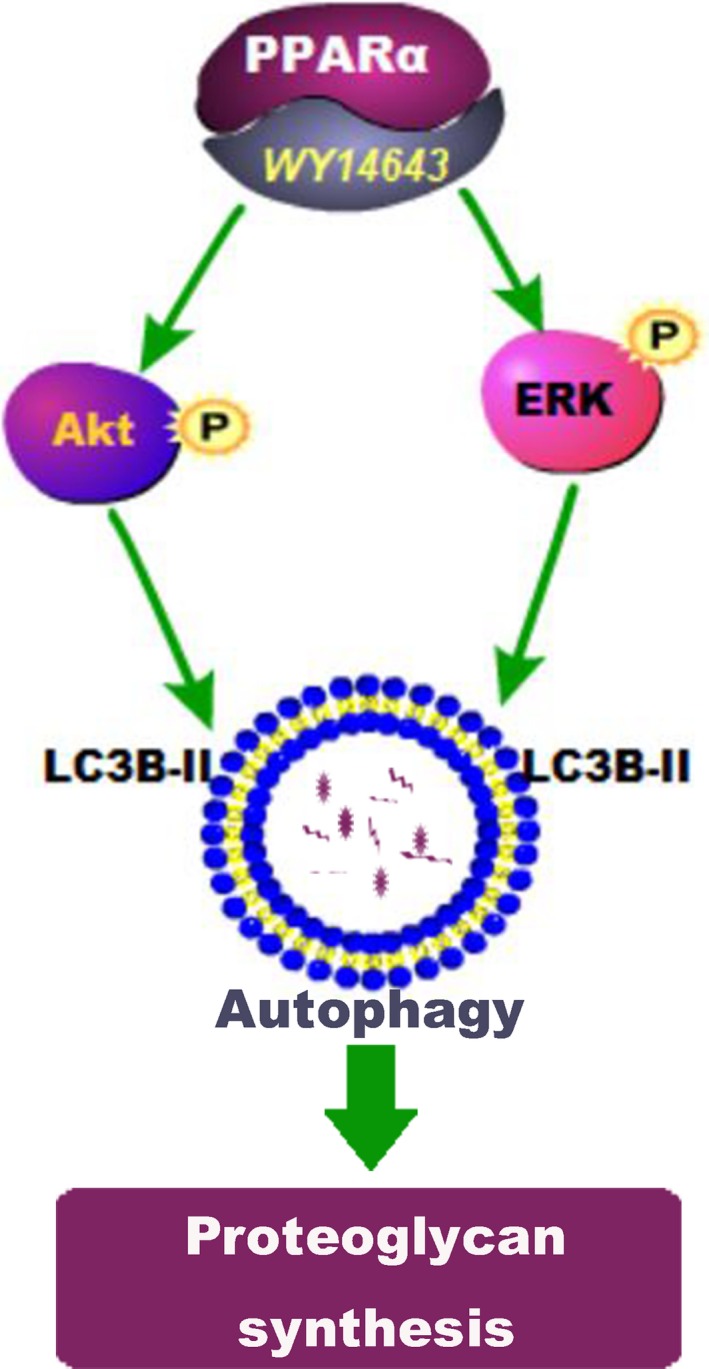
Model of PPARα activation by WY14643 promotes proteoglycan synthesis via the enhancement of autophagy in OA chondrocytes

In conclusion, PPARα activation by WY14643 could promote chondrocyte proteoglycan synthesis via the enhancement of autophagy involving Akt and ERK phosphorylation, exhibiting an effective chondroprotection on articular cartilage in OA pathological progression (Figure 7).

## CONFLICTS OF INTEREST

The authors declare that they have no competing interests.
