# Supplementary figures and images for: Chondroprotection of PPARα activation by WY14643 via autophagy involving Akt and ERK in LPS‐treated mouse chondrocytes and osteoarthritis model
Source: J Cell Mol Med. 2019 Feb 7;23(4):2782–93. doi: 10.1111/jcmm.14184 (PMC6433667; doi:10.1111/jcmm.14184)

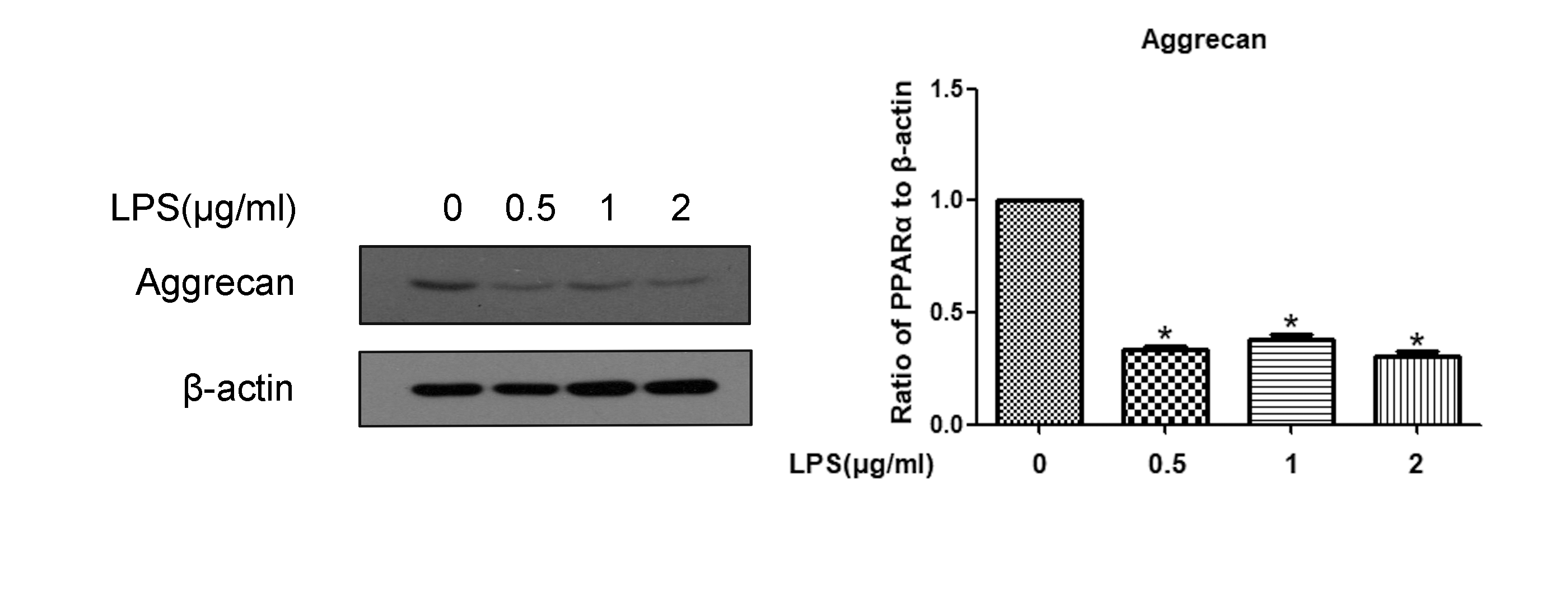

Supplement: Supplementary file 1 [file JCMM-23-2782-s001.tif]

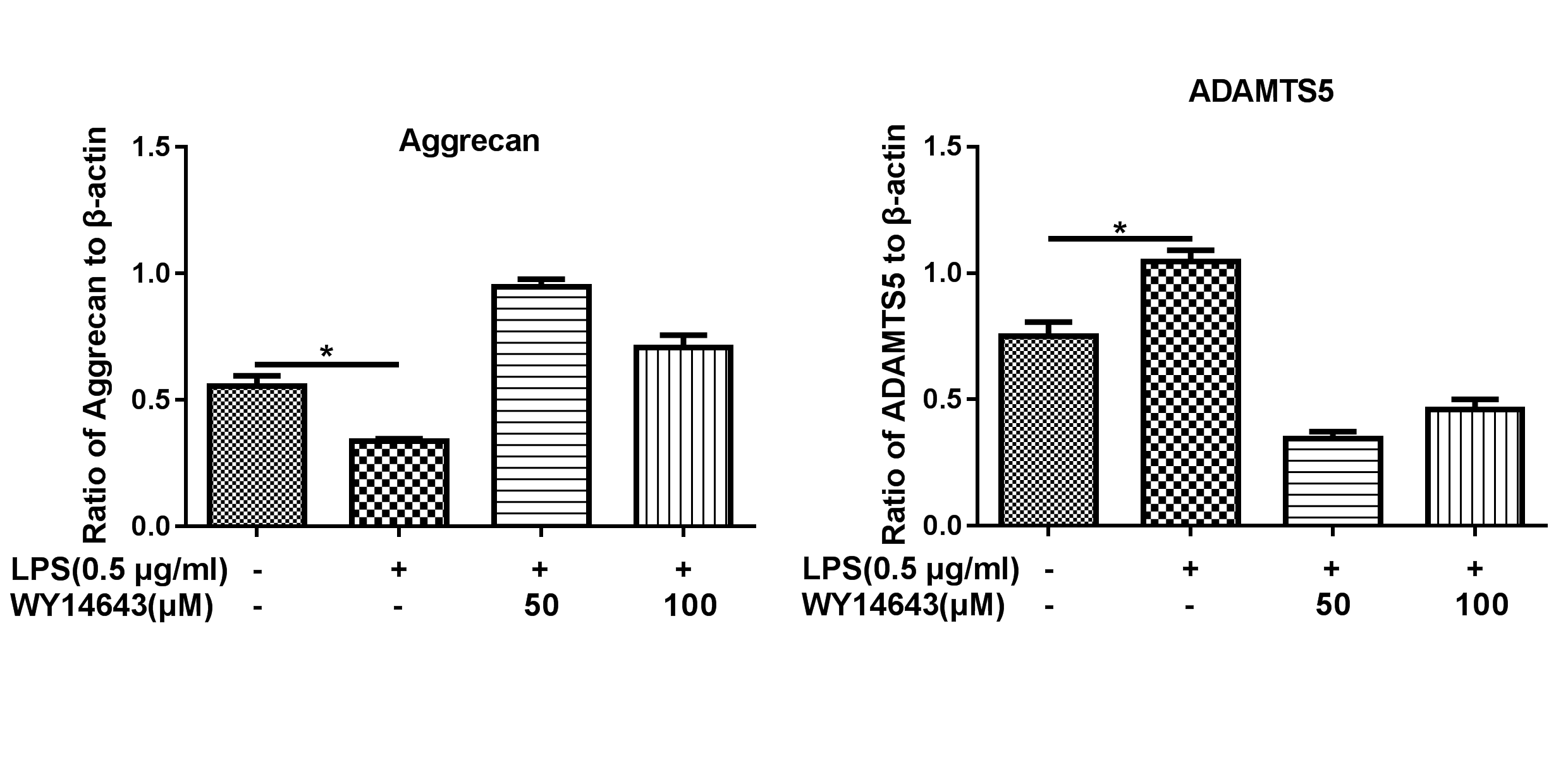

Supplement: Supplementary file 2 [file JCMM-23-2782-s002.tif]

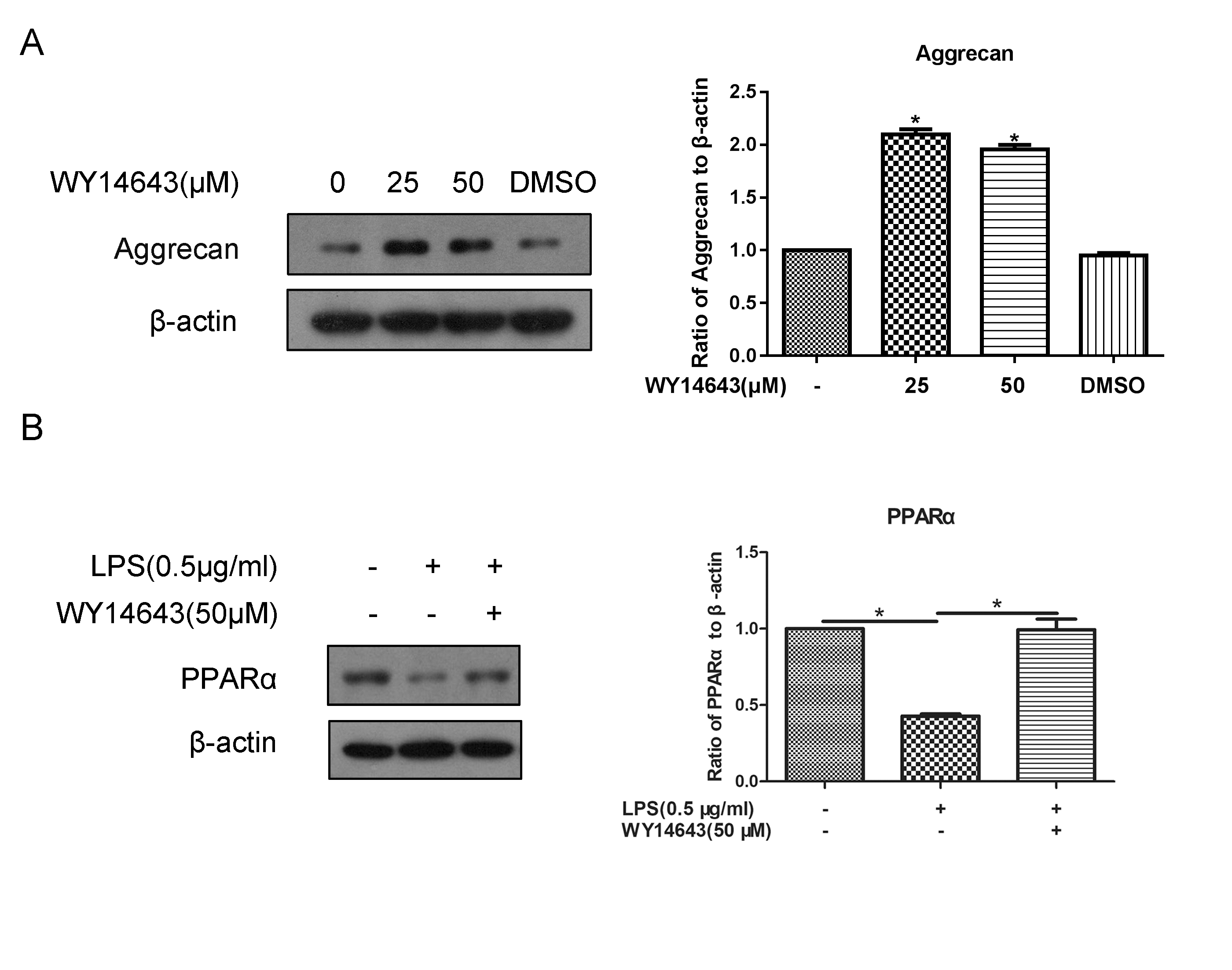

Supplement: Supplementary file 3 [file JCMM-23-2782-s003.tif]

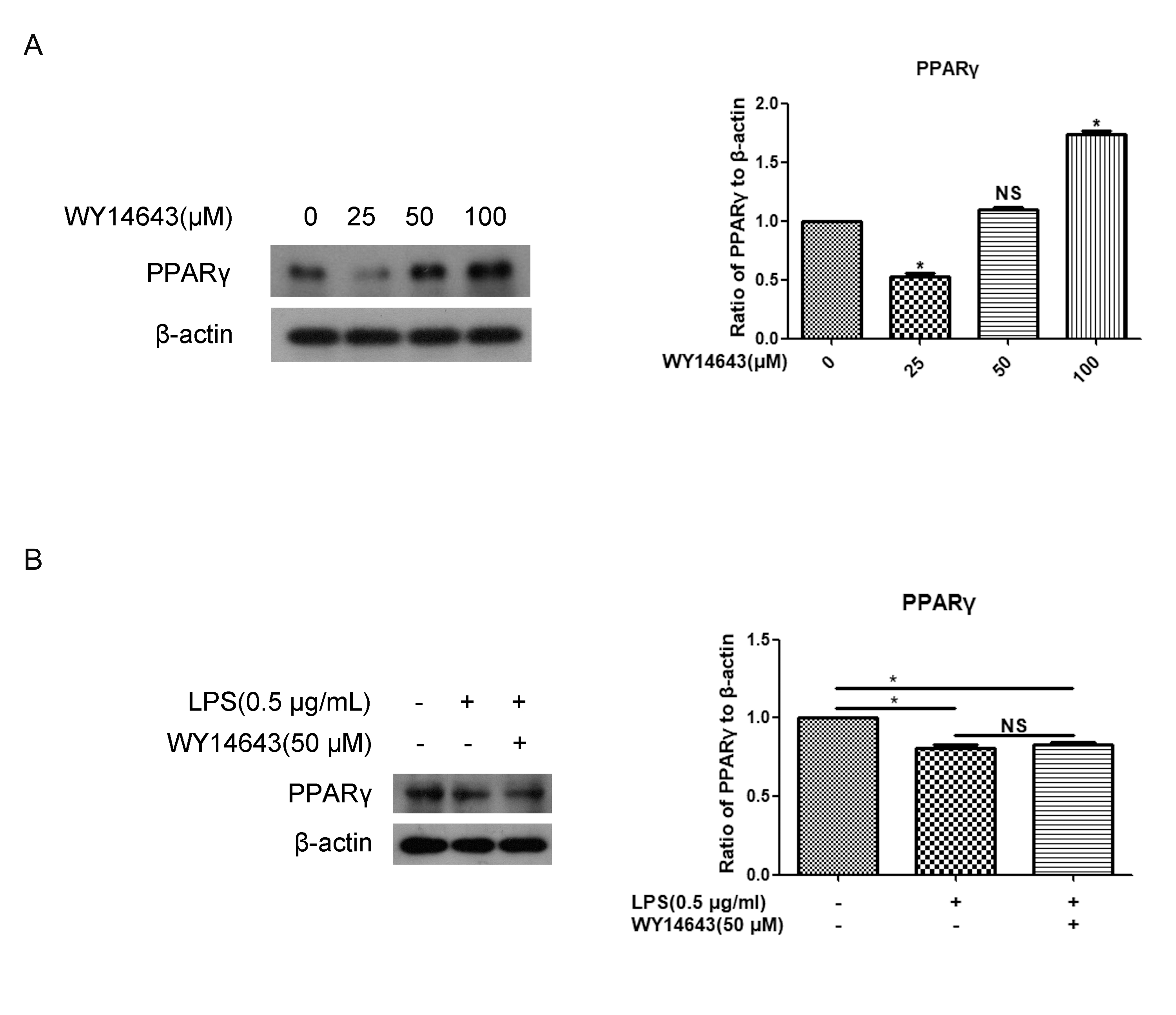

Supplement: Supplementary file 4 [file JCMM-23-2782-s004.tif]

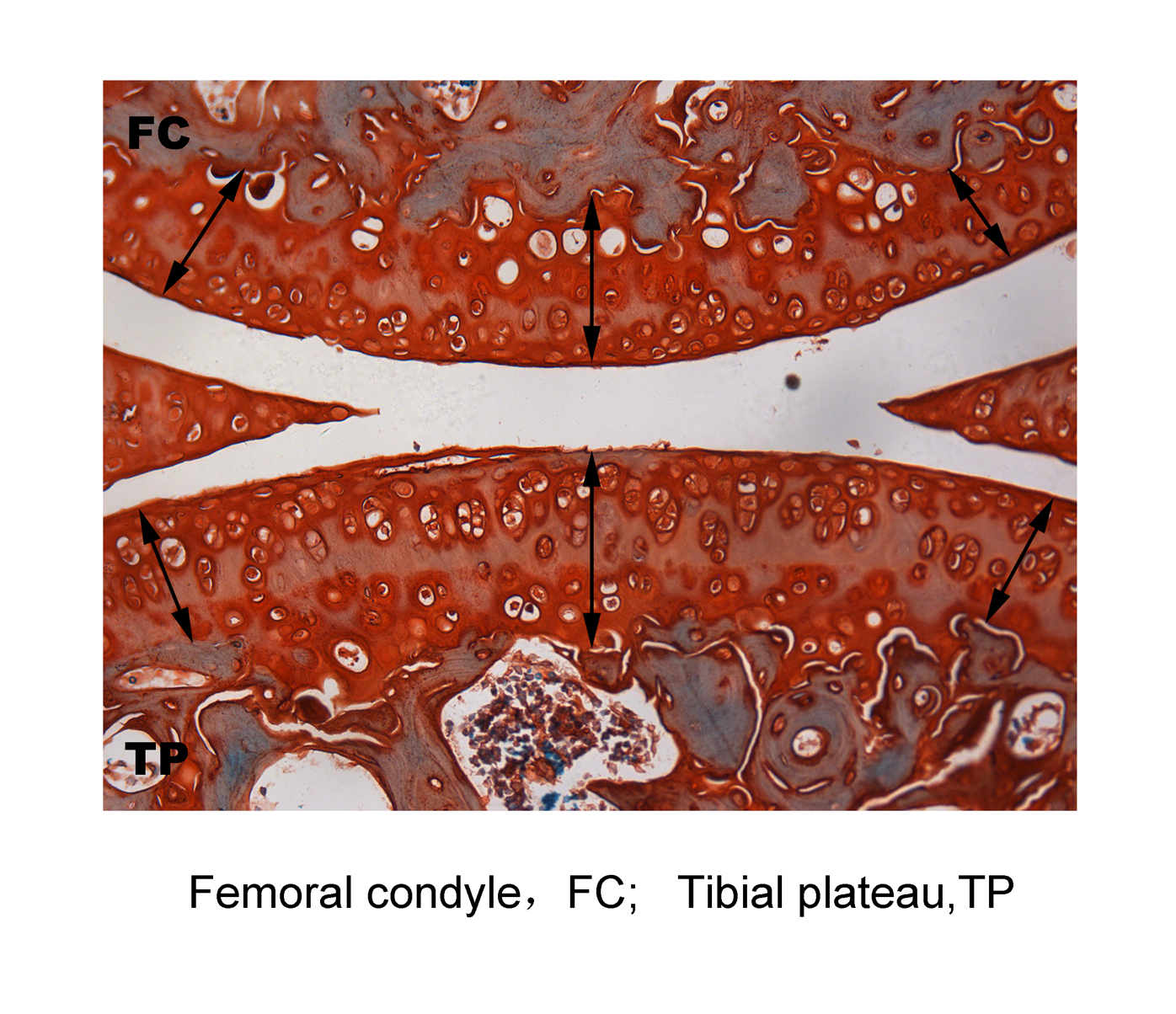

Supplement: Supplementary file 5 [file JCMM-23-2782-s005.tif]
